# Association of a single nucleotide polymorphism in growth differentiate factor 5 with congenital dysplasia of the hip: a case-control study

**DOI:** 10.1186/ar2540

**Published:** 2008-10-24

**Authors:** Jin Dai, Dongquan Shi, Pengsheng Zhu, Jianghui Qin, Haijian Ni, Yong Xu, Chen Yao, Lunqing Zhu, Hongtao Zhu, Baocheng Zhao, Jia Wei, Baorui Liu, Shiro Ikegawa, Qing Jiang, Yitao Ding

**Affiliations:** 1 The Center of Diagnosis and Treatment for Joint Disease, Drum Tower Hospital Affiliated to Medical School of Nanjing University, Nanjing 210008, Jiangsu, PR China; 2 Laboratory for Bone and Joint Diseases, Model Animal Research Center, Nanjing University, Nanjing 210008, Jiangsu, PR China; 3 Center of Diagnosis and Treatment for Congenital dysplasia of hip, Kang'ai Hospital, Nanjing 210008, Jiangsu, PR China; 4 Department of Oncology, Drum Tower Hospital Affiliated to Medical School of Nanjing University, Nanjing 210008, Jiangsu, PR China; 5 Laboratory for Bone and Joint Diseases, SNP Research Center, RIKEN, Tokyo 108-8639, Japan; 6 Department of Hepatobiliary Surgery, Drum Tower Hospital Affiliated to Medical School of Nanjing University, Nanjing 210008, Jiangsu, PR China

## Abstract

**Introduction:**

Congenital dysplasia of the hip is an abnormal seating of the femoral head in the acetabulum, mainly caused by shallow acetabulum and lax joint capsule. Genetic factors play a considerable role in the pathogenesis of congenital dysplasia of the hip. The gene growth differentiate factor 5 (*GDF5*) has been implicated in skeletal development and joint morphogenesis in humans and mice. A functional single nucleotide polymorphism (SNP) in the 5'-untranslated region of *GDF5 *(rs143383) was reported to be associated with osteoarthritis susceptibility. As a key regulator in morphogenesis of skeletal components and soft tissues in and around the joints, *GDF5 *may be involved in the aetiology and pathogenesis of congenital dysplasia of the hip. Our objective is to evaluate if the *GDF5 *SNP is associated with congenital dysplasia of the hip in people of Han Chinese origin.

**Methods:**

The *GDF5 *SNP was genotyped in 338 children with congenital dysplasia of the hip and 622 control subjects.

**Results:**

The SNP was significantly associated with congenital dysplasia of the hip (p = 0.0037; odds ration (OR) = 1.40; 95% confidence interval (CI) = 1.11 to 1.75). A significant difference was detected in female samples when stratified by gender (p = 0.0053; OR = 1.46; 95% CI = 1.21 to 1.91), and in hip dislocation when stratified by severity (p = 0.0078; OR = 1.43; 95% CI = 1.11 to 1.85).

**Conclusions:**

Our results indicate that *GDF5 *is important in the aetiology of congenital dysplasia of the hip. To the authors' knowledge this is the first time that a definite association with the congenital dysplasia of the hip susceptibility has been detected.

## Introduction

Congenital dysplasia of the hip (CDH; MIM 142700) is one of the most common congenital skeletal anomalies. CDH is an abnormal seating of the femoral head in the acetabulum [1]. CDH acts as a significant risk factor for the development of hip osteoarthritis [2-4]. Shallow acetabulum and lax capsule around the hip joint are the main causes of CDH [5,6]. Former epidemiological investigations show that CDH has a considerable genetic component. Several family studies of CDH have showed that its prevalence was significantly higher in first-degree relatives of probands [7-9]. A study of identical twins indicated hereditary factors are of prime importance in CDH [10], and a genome-wide screening of a Japanese family with acetabular dysplasia identified a linkage on a limited location of the specific chromosome [11].

Growth differentiate factor 5 (*GDF5*; also known as cartilage-derived morphogenetic protein-1) is a member of the transforming growth factor-β (TGF-β) super-family. *GDF5 *is expressed in the regions between skeletal elements where joints will later form [12,13]. It plays a crucial role in the morphogenesis of tendon, ligament and bone. A null mutation of *GDF5 *causes developmental failure of skeletal structure and intra-articular ligaments in mice [14,15]. Type C brachydactyly (MIM 113100) is a skeletal disorder caused by *GDF5 *mutation [16,17], and some patients with type C brachydactyly also present with dysplasia of hip joints [18,19].

Recently, a functional single nucleotide polymorphism (SNP) in the 5'-untranslated region of *GDF5 *(rs143383; +104T/C) was found to be significantly associated with osteoarthritis in people of Japanese and Han Chinese origin [20]. This SNP was located in the *GDF5 *core promoter and exerted allelic differences in promoter activity of the *GDF5 *gene. The susceptibility allele (+104T) showed reduced transcriptional activity of *GDF5 *in chondrogenic cells [20]. Association of this SNP with osteoarthritis has been replicated in people of European origin [21]. These findings suggest that *GDF5*, especially the functional SNP rs143383, may play a key role in the aetiology and pathogenesis of CDH. To evaluate this possible association, we examined the genetic association of the *GDF5 *SNP with CDH in people of Han Chinese origin and found a compelling association between *GDF5 *and CDH.

## Materials and methods

### Subjects

A total of 960 subjects were enrolled in this study. Three hundred and thirty-eight patients (291 females and 47 males) were enrolled consecutively at the Center of Diagnosis and Treatment for Congenital dysplasia of hip, Kang'ai Hospital, China; 622 healthy control subjects (316 females and 306 males) were enrolled at the Physical Examination Center, Drum Tower Hospital, affiliated to the Medical School of Nanjing University, China. The controls had no symptoms or histories of CDH. All subjects included in the study were of Han Chinese origin living in and around Nanjing. No subjects dropped out during the process of the study. The study was approved by the ethical committee of the participating institutions, and informed consent was obtained from patients and controls.

Patients were diagnosed by expert medical examination with radiographic evidence, and they all had unilateral or bilateral CDH. Cases with systemic syndrome were excluded from the study. Control subjects were identified by taking a detailed history and physical examination. The severity of CDH was defined from mild instability of the femoral head with slight capsular laxity, to moderate lateral displacement of the femoral head, without loss of contact of the head with the acetabulum, and then to complete dislocation of the femoral head from the acetabulum [22]. Cases were scored according to the severity of the hip disorder (1 = instability; 2 = subluxation; 3 = dislocation).

### Genotyping

DNA was obtained from all the subjects from peripheral blood using the Chelex-100 method [23] or buccal swabs using the DNA IQ System (Promega, Madison, WI) according to the manufacturer's instructions. The SNP rs143383 was genotyped using Taqman assay (Applied Biosystems 7500, ABI, Foster City, CA,. USA). Genotyping was performed by laboratory personnel blinded to case status, and two authors independently reviewed the genotyping results, data entry and statistical analyses.

### Statistics

A chi-squared test was used to compare the GDF5 genotype with the allele distributions in the case-control study. The differences in the clinical information between the genotypes were tested using the Mann-Whitney test, the Kruskal-Wallis test and the chi-squared test. The linear trend of severity was analysed by chi-squared test. Hardy-Weinberg equilibrium was performed by chi-squared test. These tests were performed using SPSS 12.0 system software (SPSS Inc., Chicago, Illinois, USA).

## Results

The ages of patients with CDH and controls (mean ± SD) were 21.6 ± 12.4 months (range 2 to 72 months) and 58.1 ± 11.0 years (range, 39 to 94 years), respectively. More than 50% of the CDH cases were delivered by caesarean section. The ratio of female to male was about six to one in patients with CDH. Distributions of genotypes in the CDH and control groups were conformed to Hardy-Weinberg equilibrium (p = 0.77 and 0.50, respectively) (Table 1). The distribution of the severity of the hip disease was 6% with score 1, 16% with score 2 and 78% with score 3 (Table 2). Significant differences in allele frequency was detected between CDH and control groups (p = 0.0037) (Table 3). Significant differences in the genotype frequency were observed in the comparison of TT (T allele homozygote) and other genotypes combined (p = 0.013), and in a comparison of CC (C allele homozygote) and other genotypes combined (p = 0.029) (Table 3). No significant difference was found between different delivery methods (p = 0.78).

**Table 1:** Genotype and allele frequencies of C/T transition SNP (rs143383) of the *GDF5 *gene in the Han Chinese population.

Group	Number of subject	Genotype (frequency)			Allele (frequency)		Hardy-Weinberg equilibrium
				
		TT	TC	CC	T	C	P value
CDH							
All	338	214 (0.633)	111 (0.328)	13 (0.039)	539 (0.797)	137 (0.203)	0.77
Female	291	185 (0.636)	95 (0.326)	11 (0.038)	465 (0.799)	117 (0.201)	0.78
Male	47	29 (0.617)	16 (0.340)	2 (0.043)	74 (0.787)	20 (0.213)	0.91
Control							
All	622	342 (0.550)	234 (0.376)	46 (0.074)	918 (0.738)	326 (0.262)	0.50
Female	316	169 (0.535)	124 (0.392)	23 (0.073)	462 (0.731)	170 (0.269)	0.97
Male	306	173 (0.565)	110 (0.360)	23 (0.075)	456 (0.745)	156 (0.255)	0.35

CDH = congenital dysplasia of the hip; GDF5 = growth differentiate factor 5; SNP = single nucleotide polymorphism.

**Table 2:** Genotype and allele frequencies of C/T transition SNP (rs143383) of the GDF5 gene in different CDH categories when stratified by severity

Group	Number of subjects (%)	Genotype (frequency)			Allele (frequency)		Hardy-Weinberg equilibrium
				
		TT	TC	CC	T	C	P value
CDH							
Instability	21 (6%)	14 (0.667)	6 (0.286)	1 (0.047)	34 (0.810)	8 (0.190)	0.74
subluxation	53 (16%)	33 (0.622)	18 (0.340)	2 (0.038)	84(0.792)	22(0.208)	0.81
Dislocation	264 (78%)	167 (0.633)	87 (0.329)	10 (0.038)	421 (0.797)	107 (0.203)	0.75

CDH = congenital dysplasia of the hip; GDF5 = growth differentiate factor 5; SNP = single nucleotide polymorphism.

**Table 3:** Association of C/T polymorphism of the GDF5 gene with CDH when stratified by gender

Groups compared	TT vs. other combined			CC vs. other combined			T allele vs. C allele			All genotype
				
	OR	P value	95% CI	OR	P value	95% CI	OR	P value	95% CI	P value
All patients (n = 338) vs all controls (n = 622)	1.41	0.013	1.08 to 1.85	0.50	0.029	0.27 to 0.94	1.40	0.0037	1.11 to 1.75	0.014
										
Female patients (n = 291) vs female controls (n = 316)	1.52	0.012	1.10 to 2.10	0.50	0.061	0.24 to 1.05	1.46	0.0053	1.21 to 1.91	0.020
										
Male patients (n = 47) vs male controls (n = 306)	1.24	0.51	0.66 to 2.33	0.55	0.42	0.12 to 2.40	1.27	0.38	0.75 to 2.14	0.65

CDH = congenital dysplasia of the hip; CI = confidence interval; GDF5 = growth differentiate factor 5; OR = odds ratio.

We stratified subjects by gender and compared the genotype distribution and allele frequency. In female samples, the most significant difference was observed in the allele frequency (p = 0.0053) (Table 3). The genotype distribution and allele frequency in male members of the CDH and control groups were similar to that in the female samples and all samples as a whole. No significant difference was detected in the comparison of genotype and allele frequency between male CDH and control subjects (Table 3). No significant difference was detected in any comparisons between female and male cases or female and male controls. A significant difference was found between samples with hip dislocation when stratified by severity (p = 0.0078) and no significant difference was found in subjects with hip instability and subluxation (Table 4). When all subjects were stratified by severity (0 = control; 1 = instability; 2 = subluxation; 3 = dislocation), a significant increasing linear trend (p = 0.020) was seen in the T allele frequency as the severity worsened.

**Table 4:** Association of C/T polymorphism of the GDF5 gene with CDH when stratified by severity

Groups compared	TT vs. other combined			CC vs. other combined			T allele vs. C allele			All genotype
				
	OR	P value	95% CI	OR	P value	95% CI	OR	P value	95% CI	P value
Patients with hip dislocation (n = 264) vs all controls (n = 622)	1.41	0.023	1.05 to 1.89	0.49	0.044	0.24 to 0.99	1.40	0.0078	1.09 to 1.79	0.028
Patients with hip subluxation (n = 53) vs all controls (n = 622)	1.35	0.31	0.76 to 2.41	0.49	0.32	0.12 to 2.08	1.36	0.22	0.83 to 2.20	0.46
Patients with hip instability (n = 21) vs all controls (n = 622)	1.64	0.29	0.65 to 4.11	0.63	0.65	0.08 to 4.77	1.51	0.30	0.69 to 3.29	0.57

CDH = congenital dysplasia of the hip; CI = confidence interval; GDF5 = growth differentiate factor 5; OR = odds ratio.

## Discussion

To the authors' knowledge this is the first demonstration of a compelling association betweenn functional *GDF5 *SNP rs143383 and CDH in the Han Chinese population. Significant differences were observed in allele frequency, and in comparisons of TT versus other genotypes combined and CC versus other genotypes combined. Significant differences were also observed in females after stratification of gender. Distribution of genotype in males was similar to that in females and the group as a whole, although no significant differences were detected in genotype and allele frequencies. No significant difference was found in any comparison between female and male subjects. The lack of significance in male subjects may be due to the limited sample number, although a large sex bias of CDH incidence obviously exists. To clarify this possible association, further research should be conducted with a larger sample number.

We discovered the significant association with hip dislocation when stratified by severity, but not with subluxation and instability. A significant increasing linear trend in the T allele frequency as the severity worsens was also observed. This indicates that the SNP may be associated with severity of CDH, but a definite conclusion could not be made because the sample number was so limited and no significant association was detected among groups of different severity.

*GDF5 *has been found to play an indispensable role in joint morphogenesis and *GDF5 *can promote the condensation of mesenchymal cells, which is the initiate step of developing cartilage element. *GDF5 *can also enhance chondrogenic differentiation of mesenchymal cells [24-28]. The T allele of rs143383 was overrepresented in CDH, and it showed a reduced transcriptional activity of *GDF5 in vitro *and *in vivo *[20,21]. Reduced expression of *GDF5 *would decrease the condensation and chondrogenic differentiation of mesenchymal cells and result in a reduction in the amount of chondrogenic cells in hip joints. It leads to a developmental deficiency of the acetabulum and proximal femoral element, especially the femoral head. As mentioned above, the absence of *GDF5 *can cause developmental failure of intra-articular ligaments in mice [14], so we suspected that a reduction of *GDF5 *expression may also lead to developmental deficiency of the ligaments and capsule in and around the human hip joint. Insufficiency of osteal elements and soft tissues in and around hip joints could contribute to susceptibility to CDH simultaneously or individually. Further study on local expression of *GDF5 *is needed to explore detailed mechanisms between reduced *GDF5 *expression and CDH.

Several association studies have been carried out to detect the susceptibility gene for CDH [29-33], and most of them produced negative results [29-31]. One study found that a *MSX1 *polymorphism was associated with limb deficiency defects including CDH [32], but it no individual data for CDH was shown. Two polymorphisms in type II collagen and vitamin D receptor genes were reported to be associated with osteoarthritis secondary to hip dysplasia [33], but another study showed a negative association of these two polymorphisms with nonsyndromic CDH [29]. Whether these two polymorphisms are associated with hip dysplasia or with osteoarthritis is still disputed. Our study is the first report of association between SNP and clearly defined CDH. Further studies should be conducted with larger sample numbers in different ethnic groups.

## Conclusions

Our study suggested that there is an association between *GDF5 *and CDH susceptibility in a Chinese Han population.

## Abbreviations

CDH: congenital dysplasia of the hip; CI: confidence interval; GDF5: growth differentiate factor 5; OR: odds ratio; SNP: single nucleotide polymorphism; TGF-β: transforming growth factor-β.

## Competing interests

The authors declare that they have no competing interests.

## Authors' contributions

All authors contributed to the final manuscript. In addition, JD and DQS genotyped the samples and participated in the design and analysis of the study. PZ, JQ, HN, YX, CY, LZ, HZ, BZ and JW evaluated the patients and genotyped these samples. BL and SI coordinated the study. QJ and YD supervised the whole study.
